# *Leishmania* RNA Virus and Its Impact on Drug Response and Clinical Outcomes in Leishmaniasis: A Comprehensive Review

**DOI:** 10.1155/japr/9924037

**Published:** 2026-05-03

**Authors:** Azadeh Zolfaghari, Sarah Seyedyousefi, Zabihollah Shahmoradi, Erfan Zaker, Bahman Rahimi Esboei

**Affiliations:** ^1^ Skin Diseases and Leishmaniasis Research Center, Isfahan University of Medical Sciences, Isfahan, Iran, mui.ac.ir; ^2^ Department of Parasitology, Toxoplasmosis Research Center, School of Medicine, Mazandaran University of Medical Sciences, Sari, Iran, mazums.ac.ir

**Keywords:** leishmaniasis, LRVs, vaccine, therapeutic approaches

## Abstract

*Leishmania* RNA viruses (LRVs) have emerged as significant modulators of disease severity, therapeutic response, and clinical outcomes in various forms of leishmaniasis. This review provides a comprehensive overview of the current knowledge surrounding LRV1 and LRV2, focusing on their taxonomy, molecular biology, epidemiological patterns, and pathogenic roles across *Leishmania* species. LRV1, predominantly infecting New World *Leishmania* (*Viannia*) species such as *L. guyanensis* and *L. braziliensis*, and LRV2, primarily associated with Old World species such as *L. major*, *L. tropica*, and *L. infantum* as viral endosymbionts, have both been associated with increased mucocutaneous dissemination, treatment failure, and disease relapse. Mechanistically, these viruses enhance parasite virulence through immune modulation—particularly via Toll‐like receptor 3 (TLR3)—leading to chronic inflammation and host tissue damage. The presence of LRV has been correlated with unresponsiveness to first‐line treatments such as meglumine antimoniate and amphotericin B, especially in endemic regions. Furthermore, LRV may serve as a prognostic biomarker, and its detection could guide therapeutic decision‐making in high‐risk patients. The review also discusses emerging therapeutic strategies targeting viral components, such as capsid proteins and RNA‐dependent RNA polymerase, as well as vaccine development using recombinant viral antigens. Finally, it emphasizes the urgent need for expanded surveillance, standardized diagnostics, and deeper exploration of host–virus–parasite interactions to better understand the clinical and epidemiological impact of LRV‐positive leishmaniasis.

## 1. Introduction

Leishmaniasis is one of the most important parasitic diseases of tropical and subtropical regions [1]. Global estimates indicate that the disease affects hundreds of thousands of people each year, representing a substantial global burden among neglected tropical diseases in terms of disease burden [2]. Clinical manifestations are highly diverse, ranging from mild skin lesions to visceral manifestations that can be life threatening if left untreated [3]. Polyvalent antimony compounds, liposomal amphotericin B, miltefosine, and some azole drugs have long been on the clinical agenda; however, treatment failure is still common, especially in northern India and South America [4]. Drug side effects, such as amphotericin‐induced renal failure and miltefosine‐induced gastrointestinal problems, further complicate the management of the disease. Researchers have been searching for new agents to explain these treatment failures [5]. One such agent is the leishmanial RNA virus, or LRV.

Researchers discovered the virus in the 1980s while studying the double‐stranded RNA in isolates of *Leishmania guyanensis*. We now know that the two main genotypes, LRV‐1 and LRV‐2, are present in different *Leishmania* species [6]. The virus belongs to the *Totiviridae* family, and its double‐stranded genome encodes two major capsid proteins and an RNA polymerase. The virus lives in the cytoplasm of the parasite and affects the interaction of the parasite with the host [7].

When the parasite carrying LRV enters a macrophage, receptors such as TLR3 and melanoma differentiation‐associated protein 5 (MDA‐5) are activated. The macrophage then secretes Type I interferon, TNF‐*α*, and IL‐6. Instead of destroying the parasite, this chronic inflammation facilitates its survival [8]. Evidence suggests that nucleotide‐binding domain, leucine‐rich repeat, and pyrin domain‐containing protein 3 inflammasome (NLRP3 inflammasome) activity is reduced in the presence of LRV, and IL‐1*β* production is reduced [9].

Animal studies showed that LRV‐positive parasites are associated with larger lesions and more widespread tissue dissemination [8]. In humans, isolates containing LRV‐1 are twice as likely to transform into the mucosal form as virus‐free isolates [10]. Patients with LRV‐positive parasites who receive amphotericin B exhibit slower recovery rates and are at higher risk of relapse [11]. The pattern of genetic distribution of LRV varies from one continent to another. Genotype 1 is prevalent in the Americas, whereas Genotype 2 has been reported in the Middle East, Central Asia, and parts of Eastern Europe. In Iran and Turkey, studies of *L. major* and *L. tropica* isolates have shown that 20%–45% of them carry LRV‐2. The lack of a standard diagnostic kit means that the true amount of virus remains unclear [12–14].

Clinical data from South America and Iran showed that LRV‐positive patients receive medication on average twice as long, have higher treatment costs, and also have higher hospitalization rates. Some studies have suggested that a combination regimen of amphotericin B and miltefosine has a better response in LRV‐positive patients, whereas amphotericin alone is not very effective [8, 15].

In practice, several key questions arise. Should LRV screening be part of the diagnostic process in endemic areas? Which laboratory method has the appropriate accuracy and cost? How can the test result be translated into clear treatment recommendations? To answer these questions, epidemiological, immunological, and pharmacological evidence must be combined into a single framework. Studies have been conducted sporadically so far, and the overall picture is still incomplete.

We also decided to take a small step, albeit one that is small, towards advancing knowledge and improving the clinical management of leishmaniasis by reviewing the literature and examining published data on the molecular biology, pathogenesis, and interaction of the LRV with the host parasite. This will help us to explain the main role of this virus in the severity of the disease and provide efficient diagnostic and therapeutic strategies.

## 2. Methods

This review was done in May 2025. Online databases, including Scopus, PubMed, and Google Scholar, were searched. The search was limited to the following query in an online search. (“Leishmania”[MeSH Terms] OR “Leishmania” OR “Leishmaniasis”[MeSH Terms] OR “Leishmaniasis” OR “Leishmania spp” OR “Leishmania infection” OR “Leishmania parasite”) AND (“Leishmania RNA virus” OR “Leishmania RNA virus 1” OR “Leishmania RNA virus 2” OR “LRV” OR “LRV1” OR “LRV2” OR “Leishmania virus” OR “dsRNA virus” OR “double‐stranded RNA virus” OR “RNA virus”).

No filter was also selected. After the search, the results were imported into EndNote software (X21 version). In the first step, the duplicated records were removed. The authors screened the records independently. The title of the records was evaluated, and irrelevant titles were excluded. In vivo and animal studies were excluded in this step. Next, the abstracts were evaluated. Those records with irrelevant content were also excluded. Finally, the authors held a meeting to merge their screening records. Then all forces were developed for finding the full text of articles. Institutional account or contact with the corresponding authors was our strategy for finding the full texts. After that, all included full texts were studied for a comprehensive review and summarization of data.

Given the aim of contextualizing mechanistic findings with clinical relevance, the review prioritized human clinical studies, epidemiological data, and translational experimental evidence. Foundational animal studies were considered when essential for mechanistic interpretation.

## 3. Results

### 3.1. Discovery, Taxonomy, and Evolution of Leishmania RNA Viruses (LRVs)

The first microscopic evidence for the presence of virus‐like particles (VLPs) in trypanosomatids dates back to 1974, with the observation of 55–60 nm structures in the cytoplasm of *Leishmania hertigi* (now called *Paraleishmania hertigi*); the particles were seen in the form of paracrystalline arrays near the kinetoplast and were stably transported during cell division. After this report, VLPs were also detected in other trypanosomatids; for example, in the monoxenous species *Angomonas* (*Crithidia*) *desouzai*, hexagonal clusters of RNA‐containing particles in the vicinity of the nucleus remained stable for 2 years in vitro culture without causing obvious cytopathic effects [16].

LRV was first discovered in 1988. Tarr et al. found it inside the parasites *L. braziliensis* and *L. guyanensis*. They saw 32‐nm round particles in the cytoplasm. Each particle held about 6000 nucleotides of single‐stranded RNA. The team decided that these particles belonged to a new RNA virus and called it LRV1 [17]. Later, other scientists—such as Stuart and Patterson—looked at the virus more closely. Their studies led to its formal placement in the *Totiviridae* family under the general name *Leishmania* RNA virus [18, 19].

The viral genome is double‐stranded RNA that measures roughly 5300 nucleotides. On the positive strand, most isolates have two main open reading frames (ORFs), ORF2 and ORF3. ORF2 codes for an 82‐kDa capsid protein, and experiments revealed that this protein can build VLPs on its own [20]. ORF3 codes for the RNA‐dependent RNA polymerase (RdRp), the enzyme the virus needs to copy its genome. The two reading frames overlap by 71 nucleotides. This small overlap holds a slippery sequence and a pseudoknot, and both features let the ribosome change‐reading frame. In test‐tube studies, the shift joins the capsid and polymerase parts into a single Cap‐Pol protein, the same trick seen in some yeast and *Giardia* viruses [21, 22].

Scientists can make Cap‐Pol in vitro, but they have not spotted it inside infected parasites. One report showed that a cysteine protease from *Leishmania* cells cuts the fusion protein into two pieces. These fragments give 82‐ and 95‐kDa bands on an SDS‐PAGE gel [22].

During the viral cycle, the RdRp enzyme copies the double‐stranded genome and produces positive‐sense RNA. Density‐gradient experiments reveal two particle types. The heavier particles contain double‐stranded RNA, and the lighter ones carry positive‐sense single‐stranded RNA that ribosomes can translate. This separation looks like what researchers have described for the yeast LAVirus, and it supports the idea that LRV follows a conservative replication plan [23, 24].

LRV RNA has another unusual trait. It lacks a 5 ^′^ cap and also skips the trans‐splicing step that most *Leishmania* mRNAs need. Instead, an internal ribosome entry site (IRES) sits at the 5 ^′^ end. One study inserted this IRES into a bicistronic DNA construct, and the element switched on the second gene. The result points to a special translation pathway in the virus [25].

For many years, scientists thought that LRV stayed quiet inside the parasite. Recent work tells a different story. Strains of *L. guyanensis* and *L. braziliensis* that carry the virus are often associated with more severe mucosal leishmaniasis and respond poorly to therapy. The current model states that macrophages notice the viral double‐stranded RNA through the TLR3 receptor. This signal raises IFN‐*β* and IL‐6 production, triggers stronger inflammation, and gives the parasite more time to survive [26].

The presence of viral particles in some *Leishmania* species was first reported in the 1980s. In 1988, Tarr et al. identified a virus called LR1 in *L. braziliensis* and *L. guyanensis*, which contained single‐stranded RNA and was present in spherical particles of approximately 32 nm [17]. A few years later, similar viruses were also discovered in other species, such as *L. guyanensis*, *L. major*, and *L. aethiopica* [27, 28]. In the 1990s, it was determined that these viruses have double‐stranded RNA and are retained within the parasite by vertical transmission (from mother cell to daughter cell). These viruses do not spread outside the cell and cannot cause independent infectivity [23]. Examination of their genomic structure showed that they contain genes for the capsid and the enzyme RdRp [29].

LRV belongs to the family of *Totiviridae* and the genus *Leishmania* virus. The genome of these viruses is a double‐stranded RNA of approximately 5300 nucleotides in length, which has two primary ORFs: one for the capsid protein and the other for the RdRp [27, 28].

In this family, most viruses are found in unicellular eukaryotic cells such as fungi, *Giardia*, and Trichomonas. Like other members of this family, LRV viruses have an icosahedral capsid and lack an envelope [30]. LRV is currently divided into two main groups. This grouping is based on gene sequences and phylogenetic relationships between the virus and its host species [31–33]. Data on host species, geographical distribution, and genetic diversity of LRV1 and LRV2 are shown in Table 1.

**Table 1:** Leishmania RNA virus (LRV) groups: Hosts, geographical distribution, and genetic diversity.

**Group**	**Host species**	**Geographical area**	**Genetic features**
LRV1	*L. guyanensis* and *L. braziliensis*	South America (New World)	High diversity (14 subgroups)
LRV2	*L. major, L. aethiopica,* and *L. infantum*	Asia, Africa, and Europe (Old World)	Less variety

Despite structural similarity, LRV1 and LRV2 differ greatly in host, genetic diversity, and impact on pathology. The details on this matter can be seen in Table 2.

**Table 2:** Comparison of biological and clinical characteristics of *Leishmania* RNA virus Type 1 (LRV1) and Type 2 (LRV2).

**Feature**	**LRV1**	**LRV2**
Host species	*Viannia* species	Old World species
Habitat	Amazon rainforest and South America	Middle East, Central Asia, and Africa
Immunological impact	Activation of the TLR3 pathway, induction of IFN‐*β*, and IL‐6	Likely weaker, still under investigation
Association with disease severity	Increased risk of mucocutaneous leishmaniasis (MCL), treatment failure	Less clearly defined, but possible effects
Genetic diversity	High (14 subtypes: LRV1‐1 to LRV1‐14)	Lower, more homogeneous
Drug susceptibility	Sensitive to 2‐C‐methyladenosine (2CMA)	Limited studies available

Research showed that LRV1, by activating the TLR3 receptor in macrophages, induces IFN‐*β* production and excessive inflammation, leading to severe tissue damage in patients with mucosal leishmaniasis [34, 35].

In contrast, knowledge regarding LRV2 is comparatively limited. Some studies have confirmed its presence in Old World species such as *L. major* and *L. infantum*, and its effect on increasing inflammatory factors has been observed in laboratory models [31, 36–38].

Phylogenetic analysis suggests that LRV viruses have coevolved with their hosts. This is reinforced by the similarity of phylogenetic trees between *Leishmania* species and their viruses. Recent studies indicated that LRV2 has three distinct lineages, found in *L. major*, *L. aethiopica*, and *Sauroleishmania*, respectively. These groups probably evolved independently and separately, and their genomic differences indicate a very old age for these viruses [30, 31, 36, 39].

### 3.2. Molecular Biology of LRV

The complete sequence of the LRV1 genome was first reported by Stuart et al. [18]. In 1992, from an isolate of *L. guyanensis*. The two ORFs overlap, and at the site of the overlap, *a* + 1 frameshift occurs, resulting in the production of the Gag‐Pol fusion protein. This frameshift is somehow similar to that observed in some retroviruses such as HIV. Such a feature probably allows the virus to be maximally efficient with minimal genetic capacity [31]. Although mechanistically analogous, LRV frameshifting is evolutionarily distinct from retroviral replication strategies. Several studies, including those by Suh and Saberi in recent years, have reported a similar structure for the LRV2 genome, which is found in Old World species such as *L. major*. These structures are highly conserved and, in phylogenetic analyses, are closely related to viruses of the *Totiviridae* family from an evolutionary perspective [27, 28].

A distinctive feature of the LRV capsid is the presence of an endoribonuclease with a highly specific cleavage in the 5 ^′^‐UTR regions of the genome. Mutagenesis microdynamics indicate that the minimum sequence required for recognition of the enzyme is in the range of nucleotides 249–342; in this interval, there is a 40‐nucleotide hairpin structure, the deletion or disruption of which completely blocks cleavage. The endoribonuclease activity is only carried out in the presence of divalent cations such as Mg^2+^ and Ca^2+^, and salt concentrations above 25 mM inhibit it. Supplementary data show that the increase in the number of assembled virions correlates with an increase in the frequency of cleavage and a decrease in the efficiency of viral RNA translation [40].

As the number of LRV virions increases in the cytoplasm, the capsid endoribonuclease enters a negative feedback loop; the enzyme cleaves not only viral transcripts but also parts of *Leishmania* 18S rRNA at sequences similar to the viral cleavage site. This cleavage produces short 5 ^′^ fragments that are unable to bind to ribosomes, thereby reducing the overall rate of cellular translation. The reduction in translation reduces the metabolic stress caused by the high production of viral proteins and allows the virus to remain at a stable, low‐copy level. Grybchuk et al. [16], colleagues have considered this mechanism as an example of long‐term adaptation of the virus to the host, since the delicate inhibition of the translation machinery maintains a balance between parasite survival and cellular survival.

Unlike many messenger RNAs in *Leishmania*, the LRV viral RNA lacks a splice leader sequence. The 5 ^′^ region of the LRV genome is approximately 450 nucleotides long and does not encode a protein, but it appears to be involved in RNA stability and translation initiation [41]. In Stuart et al. [18], this region is described as a conserved region that is found in all LRV subspecies. Sequence analysis suggests that the secondary structure of the RNA in the 5 ^′^ region may contribute to the formation of specific protein complexes with the RdRp. Also, repetitive sequences have been found in the 3 ^′^ end of the genome that may play a role in determining the length of the new RNA. Such features allow the virus to replicate within the parasite without relying on host components for RNA processing. This independence provides a high degree of adaptation to specific conditions within the parasite cells, which is not observed in many endosymbiotic viruses [27, 28, 31].

Suh et al. [28] showed that the RdRp stays active inside the viral capsid and copies RNA straight from the double‐stranded template. The reaction skips both an mRNA step and any DNA intermediate. ORF3 supplies the code for RdRp, and the virus usually translates this gene along with the capsid gene to create one Gag‐Pol fusion protein. This layout keeps replication tightly controlled inside the small capsid and helps protect the genome. New work on LRV2 compared RdRp genes from several lineages and found sequence changes in the parts that are not conserved [31].

According to microscopic studies by Soares et al. [32], LRV virions accumulate mostly around the nucleus of *Leishmania* cells and close to the endoplasmic reticulum and mitochondria. Such a location facilitates the simultaneous translation of viral RNAs and faster assembly of virions. In a paper by Valencia et al. [42], it was observed that within some isolates of *L. braziliensis*, viral capsids accumulated next to ribosomal granules, indicating active translation. This suggests that the location of capsid accumulation is likely directly related to the process of RNA replication and translation. In the study by Telittchenko and Descoteaux [43], which focused on genetic exchange in *Leishmania*, it was observed that the area of virus accumulation may also be associated with the accumulation of specific genes, and for this reason, the structural role of viral RNAs was also considered [44].

Mutagenesis studies exhibited that the 5 ^′^‐UTR of the LRV genome contains a hairpin structure of approximately 40 nucleotides that acts as a “density sensor.” When the number of VLPs is low, the IRES of this region allows full‐length transcripts to be translated with high efficiency. However, as the number of virus particles increases, the capsid protein cleaves this loop at a specific site; the cleavage splits the RNA into two 5 ^′^ and 3 ^′^ fragments, and the short 5 ^′^ fragment competes with the long transcript for binding of initiation factors. The result is a decrease in the rate of viral protein synthesis and inhibition of excessive replication; a negative feedback mechanism that maintains virion numbers at a stable level compatible with parasite survival [40].

Structural and molecular characteristics of LRV1 and LRV2 and their differences can be seen in Table 3.

**Table 3:** Structural and molecular characteristics of LRV1 and LRV2 and their differences.

**Feature**	**LRV1**	**LRV2**
Genome length	~5280 nucleotides	Similar (±50 nt)
Number of ORFs	3 (including two major ORFs)	2–3, similar
Position of ORF2 and ORF3	Overlapping with +1 frameshift	Similar
Capsid	Icosahedral, approximately 40–50 nm	Similar
Envelope	Absent	Absent

### 3.3. Epidemiology and Global Distribution

LRVs, particularly LRV1 and LRV2, have been receiving increasing attention due to their involvement in the pathogenesis and severity of leishmaniasis, as well as their effect on the patients′ responses to treatment. LRV1 has been mostly associated with *Leishmania* (*Viannia*) species, including *L. braziliensis*, *L. guyanensis*, and *L. panamensis*, and has been detected in a number of endemic areas of Brazil, Peru, Colombia, and Panama [45–49]. Prevalence rates vary in different studies, ranging from 20% to over 70%, depending on the species and local epidemiology [46, 47, 49, 50]. For instance, in French Guiana and northeastern Brazil, LRV1‐positive isolates have been linked to severe or mucosal forms of tegumentary leishmaniasis [50–52]. LRV1 is clinically significant because it is often linked to mucosal involvement and a poor response to treatment [47, 51, 53–55]. Additionally, its presence across distinct parasite isolates within individual patients underscores its potential role in driving intrahost variability, suggesting broader implications for disease progression and therapeutic challenges [53, 56].

LRV2 has been reported to be less prevalent than LRV1; however, some studies have reported detection rates above 20% [57–59]. LRV2 has been linked to lesion exacerbation, delayed healing, and treatment unresponsiveness, particularly in *L. major*‐infected patients [14, 27, 31, 58, 60].

The global distribution of LRVs has a specific geographic pattern. LRV1 is mainly limited to the New World and species of the *Leishmania* (*Viannia*) subgenus [45–50]. Studies in Argentina, Panama, and Colombia have demonstrated varying LRV1 prevalence among *L. braziliensis*, *L. panamensis*, and *L. guyanensis* isolates, but with interspecies and geographical differences [30, 45, 46, 48]. Furthermore, genetic studies confirm that New World LRV1 strains exhibit distinct evolutionary lineages that are associated with geographic origin [6, 61]. Interestingly, a case of *L. aethiopica* from Ethiopia was found to harbor a unique LRV1 strain, expanding the known host range beyond the *Viannia* subgenus [62].

On the other hand, LRV2 has been seen in *L. major* from Iran, Turkey, and Uzbekistan [38, 57, 58, 63, 64], and recently in *L. infantum* strains from both human and canine visceral leishmaniasis cases in Brazil [65, 66]. The detection of LRV2 in both zoonotic (*L. infantum*) and anthroponotic (*L. major*) species suggests its epidemiological importance [65, 66]. Cocirculation of these viruses raises important questions about their transmission patterns, evolutionary dynamics, and species‐specific interactions with different *Leishmania* hosts [14, 67].

A summary of LRV distribution and prevalence is shown in Table 4.

**Table 4:** Leishmanial RNA viruses′ distribution and prevalence around the world.

	**LRV type**	**Country/region**	**Reported prevalence**	**Reference**
*L. guyanensis*	LRV1	French Guiana	38%–92%	[51, 52]
*L. braziliensis*	LRV1	Brazil and Peru	20%–50%	[46, 49]
*L. panamensis*	LRV1	Colombia and Panama	15%–40%	[30, 48]
*L. major*	LRV2	Iran, Turkey, and Uzbekistan	10%–25%	[14, 38, 57, 58, 63]
*L. infantum*	LRV2	Brazil (human and canine)	15%–20%	[65, 66]
*L. aethiopica*	LRV1	Ethiopia	Case report	[62]

However, it is worth mentioning that reported prevalence varies substantially depending on detection method (RT‐PCR vs. nested PCR vs. IFA), limiting cross‐study comparability.

### 3.4. Pathogenesis: How LRV Modulates *Leishmania* Virulence

The double‐stranded nature of the viral RNA allows it to be recognized by TLR3 in the endosome of macrophages. In a mouse model of LRV‐positive *L. guyanensis,* this TRIF‐mediated recognition activates the secretion of TNF‐*α*, IL‐6, and Type I interferon and induces severe inflammation at the site of infection; whereas deletion of LRV or the use of TLR3‐/‐mice inhibits these effects. Lesion severity, parasite burden, and metastasis to distant tissues have been reported to be significantly higher in animals infected with this viral strain [68]. In addition to the dominant TLR3 pathway, in vitro data indicate that LRV transcripts or single‐stranded fragments can also mildly stimulate the endosomal receptor TLR7 and induce limited levels of inflammatory cytokine secretion in mouse macrophages; however, genetic deletion of TLR7 in the C57BL/6 model was not associated with any difference in lesion size or parasite burden compared with wild‐type mice. Thus, a weak TLR7 response alone is not sufficient for clinical exacerbation, and the regulation of pathology appears to remain largely under the control of TLR3 signaling. The double‐stranded nature of the viral RNA is not limited to TLR3; the same molecule can also be recognized by the cytosolic receptors RIG‐I and MDA‐5 and possibly by NLR pathways. These pathways may contribute to tissue destruction by producing IFN‐*β* and activating the inflammasome, although the exact magnitude of their effect in metastasis models is still unclear. This latent population is reactivated and metastasizes under conditions of immunosuppression or secondary stress, a phenomenon that highlights the importance of long‐term patient monitoring. Some LRV^+^ isolates also elevate insulin‐like growth factor (IGF) levels. IGF induces the expression of angina I and, by consuming arginine, reduces the production of toxic nitric oxide; as a result, the parasite escapes the macrophage′s lethal response and has the opportunity to spread [8].

Despite the strong activation of the antiviral response, the presence of LRV paradoxically suppresses the activation of the NLRP3 inflammasome and the secretion of IL‐1*β*. In human specimens, mucocutaneous lesions infected with LRV+ exhibit lower levels of IL‐1*β* and activated caspase‐1 than LRV‐ lesions; an effect that is associated with an increased risk of metastasis. The mechanism of this suppression in murine macrophages involves the induction of autophagy and the destruction of the NLRP3‐ASC complex by TLR3 signaling. Reduction of IL‐1*β* not only rescues the parasite from nitric oxide‐dependent death but also inhibits uncontrolled destructive inflammation and leads to long‐term parasite survival [68, 69].

The early peak of Type I interferon (particularly IFN‐*β*), which is induced in macrophages after recognition of LRV double‐stranded RNA via TLR3, stimulates the secretion of a group of neutrophil chemokines, such as CXCL10 and CCL4. This IFN‐I–chemokine axis, by increasing the recruitment of neutrophils to the lesion bed, creates an inflammatory environment that not only does not lead to parasite clearance but also contributes to tissue destruction and wound chronicity by the continued presence of multinucleated cells and the production of oxygen radicals. In LRV^+^ mouse models, inhibition of the IFN‐I receptor (IFNAR) reduced the lesion and parasite burden; instead, neutrophils accumulated more persistently at the site of infection, and their killing efficiency increased. This finding suggests that virulent IFN‐I, while attracting neutrophils, impairs their intrinsic antileishmanial function, thus leading to long‐lasting but ineffective inflammation [70].

In C57BL/6 mice infected with the *L. guyanensis* LRV+ clone, lesion diameter, parasite burden in the skin and lymph nodes, and tissue involvement are much greater than in animals infected with the same strain without the virus. Even after the virus is removed from the parasite, the rate of replication in culture is unchanged, but the pathogenicity in the animal is reduced [68]. In the early 2010s, several reports suggested an association between LRV1 and mucocutaneous and treatment resistance. However, a 24‐month cohort study of 56 Peruvian patients showed that the prevalence of LRV1 was not significantly different between metastatic (40%) and nonmetastatic (58%) phenotypes; treatment failure rates were also similar in LRV+ and LRV− patients. The authors concluded that although LRV could be a risk factor, it alone does not determine disease outcome and that the interaction of parasite species, parasite load, host immunity, and comorbidities should be considered [71, 72].

Researchers in Bolivia and Brazil report that *Leishmania brasiliensis* strains that carry LRV1 are associated with more relapses and respond poorly to treatment. In French Guiana, however, mucositis appears only occasionally, even though the virus is common [54]. The most likely explanation is variation in parasite genes, host genes, viral burden, and the diagnostic tests in use.

Three proteins—GP63, HSP90, and MPI—have key roles: They let the parasite survive in macrophages, evade the complement system, and build its surface glycoconjugates. In a set of 24 South‐American isolates, scientists found slightly higher expression of mpi and hsp90 in parasites without the virus, whereas GP63 levels stayed about the same in both groups [71].

IL‐1*β* suppression facilitates parasite survival on the one hand, and on the other hand, paves the way for a secondary cytokine burst. In the human model, LRV‐positive patients, although they have less IL‐1*β* in the lesion, have similar TNF‐*α* concentrations to LRV‐; that is, a “low‐grade, chronic” inflammation replaces the acute burst, paving the way for parasite dissemination [73].

### 3.5. Impact of LRV on Antileishmanial Drug Response, Clinical Manifestations, and Outcomes

Multiple studies demonstrated a correlation between the presence of LRVs and the failure of standard antileishmanial therapies. *Leishmania* (Viannia) *braziliensis* and *L. guyanensis* are examples of this drug unresponsivity, particularly in patients from Peru, Bolivia, and French Guiana [11, 47]. In these cases, treatment was primarily based on pentavalent antimonials and amphotericin B.

Similarly, in Iran, clinical isolates of *L. major* harboring LRV2 were associated with unresponsiveness to meglumine antimoniate [14, 60, 74]. Affected patients experienced persistent lesions or progression even after receiving standard dosages of intralesional or systemic antimonials. This suggests that the virus may affect parasite susceptibility or host immune reactions.

In addition to drug‐specific effects, LRV1 has also been shown to hijack the parasite′s exosomal pathway. It might facilitate the release of viral material within exosomes that are taken up by host macrophages [75]. This exosomal transmission enhances immune evasion and might contribute to reduced drug sensitivity by changing host inflammatory signaling.

LRV1 has also been associated with increased risk of symptomatic relapse and chronic disease. For instance, LRV1‐positive isolates were found to be more likely to be involved in relapses among patients who were infected with *L. guyanensis* in French Guiana. It is worth mentioning that this relapse was seen after apparent clinical cure following treatment with meglumine antimoniate [47]. The virus′s persistence in tissues and promotion of inflammation may be the reason for these relapses.

In a study from Colombia, patients who developed mucosal leishmaniasis were significantly more likely to have been infected with LRV1‐positive *Leishmania* strains or to have a history of cutaneous leishmaniasis that was not adequately treated with pentavalent antimonials alone or to have received amphotericin B too late in the disease course. This finding supports the role of LRV1 in promoting disease progression from cutaneous to mucosal forms when early and effective treatment is not administered [55].

However, there are inconsistencies on this matter in the present literature. *L. braziliensis* strains harboring LRV1 were not always associated with worse outcomes following pentavalent antimonial or liposomal amphotericin B treatment. This suggests that viral influence may be strain‐specific or moderated by host factors [76].

This context‐dependent interpretation is further supported by recent experimental evidence showing that although LRV‐1 enhances infectivity and modulates macrophage cytokine responses in vitro; its presence alone does not consistently correlate with severe clinical outcomes or treatment failure across *Leishmania* (*Viannia*) species, particularly in *L.* (*V.*) *panamensis* [77].

Considering the available results on treatment resistance and disease severity, the presence of LRVs should be investigated as a prognostic biomarker. Several studies advocate for pretreatment viral screening in endemic settings to identify patients at risk for poor therapeutic response [11, 14, 55, 74].

In one remarkable case report, treatment failure occurred in a patient infected with *L.* (*Viannia*) *naiffi* harboring LRV1, despite adequate treatment. This underscores the potential clinical importance of detecting even rare LRV‐parasite pairings [66].

The presence of *Leishmania* RNA virus, particularly LRV1, has been associated with more severe clinical manifestations, including mucosal involvement, atypical lesions, and increased inflammatory response. Patients infected with *L.* (*Viannia*) *braziliensis* or *L. guyanensis* strains harboring LRV1 are at greater risk of developing mucocutaneous leishmaniasis (MCL), especially in endemic areas of Brazil, Peru, and French Guiana [50, 51, 78]. In one study, LRV1 was found in over 60% of *L. braziliensis* isolates from mucosal cases, compared with lower rates in cutaneous forms [51].

LRV1 not only affects disease severity but also the pattern and location of lesions. Studies exhibited that LRV1‐positive strains may contribute to more diffuse or ulcerative lesions, delayed healing, and nasal mucosal damage, particularly in *L. braziliensis* cases [51, 71, 78, 79].

Furthermore, *L. panamensis* isolates from Colombia showed low to undetectable LRV1 prevalence, as well as lower rates of mucosal complications, emphasizing LRV‐related pathogenicity [30].

Interestingly, although several studies confirm the link between LRV1 and more severe clinical outcomes, a Brazilian cohort has reported that LRV1 presence did not consistently predict worse clinical outcomes, with some patients responding well to standard therapies despite LRV positivity [76]. These findings suggest that host genetic factors, coinfections, or parasite strain diversity may mediate the virus′s impact on disease expression.

The immunopathology associated with LRV is thought to be related to its ability to stimulate pro‐inflammatory pathways, especially through TLR3 signaling, leading to exaggerated immune responses that damage host tissues [37, 80, 81]. This excessive inflammation has been implicated in the development of mucosal forms and treatment‐resistant lesions, even when the parasite burden is low.

If LRVs prove to be significantly effective on the prognosis, as molecular diagnostic tools become more accessible, integrating LRV screening into routine workflows might improve clinical outcomes by enabling personalized treatment strategies for high‐risk patients. Also, it is suggested that LRV1 may upregulate virulence‐related genes, potentially altering the parasite′s ability to persist and spread within host tissues [37, 56, 80].

Collectively, the clinical presentation of LRV‐positive leishmaniasis can reflect a complex interaction between viral presence, *Leishmania* species, host immunity, and treatment protocols. Additional molecular and immunological studies are needed to define which patients are most likely to relapse, develop chronic infection, and experience mucosal dissemination. This can help us modify and plan better treatment protocols to effectively treat the patients. A summary of LRV effects on lesions′ prognosis and relapse is shown in Table 5.

**Table 5:** The effects of LRV on the treatment progression of *Leishmania* lesions.

**Species**	**LRV type**	**Country/region**	**Treatment used**	**Reported outcome**	**Significance reported**	**Reference numbers**
*L. guyanensis*	LRV1	French Guiana	Meglumine antimoniate	Symptomatic relapse after clinical cure	Yes	[47]
*L. braziliensis*	LRV1	Brazil and Peru	Meglumine antimoniate and amphotericin B	Poor response and mucosal progression	Yes	[11, 50, 51]
*L. major*	LRV2	Iran	Meglumine antimoniate	Persistent lesions and delayed healing	Partial	[14, 60, 74]
*L.* (*V.*) *naiffi*	LRV1	Brazil	Meglumine antimoniate and amphotericin B	Treatment failure despite standard therapy	Case report	[66]
*L. braziliensis*	LRV1	Brazil	Pentavalent antimonials and liposomal amphotericin B	Mixed results: some patients with LRV1 responded well	No	[76]
*L. panamensis*	LRV1	Colombia	Pentavalent antimonials	Lower prevalence of LRV1 and fewer mucosal cases	Not applicable	[30]

### 3.6. Diagnostic Approaches for Detecting LRV

The gold standard for virus detection is still reverse transcription polymerase chain reaction (RT‐PCR). Guiana, Ginouvès et al. [50] used a 124‐bp primer on the RdRp gene and found LRV1 in *L. guyanensis* cultures. They then amplified the 779‐bp fragment to determine the genotype. Researchers in Iran tested the same method in a seminested manner and showed the presence of LRV2 in two isolates of *L. major* and *L. infantum* [73].

To isolate the virus in bulk, early researchers tried to extract dsRNA; they digested single‐stranded RNA with RNase A in high salt concentration and observed a 5.3 kb band size, then concentrated the particles with a CsCl gradient and measured RdRp activity. This method is still used in laboratories without sequencing facilities [82, 83].

In the Middle East, indirect IFA is recommended as a field screening method. Hajjaran et al. [84] injected whole virus antigen, produced polyclonal antibodies, and identified four *L. major* LRV2^+^ isolates by fluorescence microscopy; the results were consistent with seminested RT‐PCR. IFA does not require purified RNA and can be performed in field laboratories.

At present, LRV detection should be regarded as an investigational tool rather than a validated prognostic biomarker. The absence of standardized assays, clinically meaningful viral load thresholds, and prospective multicenter validation limits its immediate applicability in routine diagnostic workflows, particularly in resource‐limited endemic settings.

### 3.7. Therapeutic and Vaccine Perspectives

Considering the role of LRV in *Leishmania* pathogenesis and its contribution to treatment failure, some studies have explored LRV‐specific targets as a novel therapeutic strategy. Characterization of the viral RdRp and capsid proteins, which are essential for viral replication and stability, helped significantly in this matter [17]. These components serve as attractive antiviral targets.

Some adenosine analogs, such as 2 ^′^C‐methyladenosine (2CMA), selectively inhibit LRV1 polymerase activity and reduce viral load without directly affecting the parasite itself [35, 85, 86]. These findings are promising as they offer a strategy to decrease the virulence‐enhancing effects of the virus without inducing drug resistance in *Leishmania*.

Moreover, LRV suppression by capsid protein overexpression or by selection using hygromycin B has proved useful in virus removal from infected parasites, but the procedures are best utilized in the laboratory and potentially are not clinically translatable or safe [87].

Beyond antivirals, LRV also presents a vaccine target—either through the virus itself or by modulating the host immune response it dysregulates. A seminal study demonstrated that immunization with recombinant LRV1 capsid protein in mice significantly reduced disease severity in LRV1‐positive *Leishmania*, likely by promoting neutralizing immunity against the viral component [54]. This strategy could form the basis for adjunct or pan‐*Leishmania* vaccines, particularly in areas where LRV‐positive strains are endemic.

The immunological rationale behind this approach is supported by evidence that LRV acts as a pathogenic cofactor, skewing immune responses via TLR3 activation. A vaccine that blocks or preconditions the host to tolerate or neutralize viral components could blunt the excessive inflammation seen in mucocutaneous or treatment‐resistant disease forms.

Recent research also suggests that eliminating LRV from parasites can significantly alter parasite phenotype and infectivity, further validating LRV as a therapeutic target. In a study involving the CRISPR/Cas9‐based elimination of LRV from multiple *Leishmania* species, viral clearance resulted in reduced inflammation and slower disease progression [88]. However, the species‐specific variability in response to viral elimination indicates that not all *Leishmania*‐LRV pairings produce identical immune or pathogenic outcomes.

As antiviral strategies mature, challenges remain regarding target specificity, delivery, and resistance prevention. Nonetheless, the concept of combining traditional antileishmanial drugs with LRV‐targeting antivirals or vaccines holds great potential for improving outcomes, especially in patients with persistent or mucosal disease.

## 4. Future Perspective

Despite significant progress in understanding the role of LRVs in disease pathogenesis and treatment response, substantial gaps remain that warrant focused investigation. First and foremost, there is a pressing need for comprehensive studies on LRV genetic and functional diversity, particularly across lesser‐studied geographic regions and *Leishmania* species. Much of the current literature focuses on *L. braziliensis* and *L. guyanensis*, whereas the diversity and biological behavior of LRV1 and LRV2 in other species, such as *L. major*, *L. aethiopica*, and *L. infantum*, remain poorly characterized [89].

To refine our understanding of LRV‐associated pathogenesis, it is critical to investigate the species–specific interactions between LRVs and their *Leishmania* hosts. Evidence suggests that the impact of the virus on disease progression may vary widely depending on parasite species, host immune context, and viral genotype [90, 91]. For instance, although LRV1 in *L. guyanensis* has been linked to enhanced metastasis and immune‐mediated tissue damage, similar associations in other *Leishmania* species are either weaker or absent, highlighting the need for strain‐resolved studies and comparative genomics.

Equally important is the exploration of LRV′s role in modulating host immunity. The virus is known to engage pattern recognition receptors (PRRs) such as TLR3, triggering a cascade of Type I interferon responses that paradoxically promote parasite persistence through immune dysregulation [90, 92]. These pathways—originally evolved to defend against viral infections—are subverted by LRV to create a permissive environment for chronic *Leishmania* infection. However, the precise cellular and molecular mechanisms underlying this immune manipulation remain to be fully elucidated.

Additionally, the broader implications of microbial coinfections, viral evolution, and host–pathogen–virus triadic interactions must be integrated into future studies. With increasing evidence that endosymbiotic viruses like LRV can influence virulence, immune evasion, and therapeutic outcome, the *Leishmania*‐LRV model represents an ideal system to explore microbial synergy in parasitic diseases [89, 91].

Looking ahead, the development of pan‐*Leishmania* diagnostic panels that include LRV detection, as well as the integration of LRV surveillance into epidemiological and therapeutic studies, will be essential. These strategies could not only help tailor treatment approaches but also pave the way for host‐ and virus‐targeted interventions aimed at mitigating the clinical burden of LRV‐positive leishmaniasis.

## Funding

No funding was received for this manuscript.

## Consent

The authors have nothing to report.

## Conflicts of Interest

The authors declare no conflicts of interest.

## Data Availability

The data that support the findings of this study are available from the corresponding author upon reasonable request.
